# Research progress on the inhibition of oxidative stress by teriparatide in spinal cord injury

**DOI:** 10.3389/fneur.2024.1358414

**Published:** 2024-04-22

**Authors:** Gangtong Ai, Moliang Xiong, Liang Deng, Jihuan Zeng, Qiang Xiao

**Affiliations:** ^1^Department of Jiangxi Medical College, Nanchang University, Nanchang, Jiangxi, China; ^2^Department of Orthopaedics, Jiangxi Provincial People's Hospital, The First Affiliated Hospital of Nanchang Medical College, Nanchang, Jiangxi, China

**Keywords:** spinal cord injury, oxidative stress, Nrf2, teriparatide, PTH

## Abstract

Spinal cord injury (SCI) is currently a highly disabling disease, which poses serious harm to patients and their families. Due to the fact that primary SCI is caused by direct external force, current research on SCI mainly focuses on the treatment and prevention of secondary SCI. Oxidative stress is one of the important pathogenic mechanisms of SCI, and intervention of oxidative stress may be a potential treatment option for SCI. Teriparatide is a drug that regulates bone metabolism, and recent studies have found that it has the ability to counteract oxidative stress and is closely related to SCI. This article summarizes the main pathological mechanisms of oxidative stress in SCI, as well as the relationship between them with teriparatide, and explores the therapeutic potential of teriparatide in SCI.

## Introduction

Spinal cord injury (SCI) refers to the varying degrees of damage to the spinal cord caused by various etiologies. It can be classified into primary injury and secondary injury. Primary injury refers to the initial acute trauma that causes damage to the neural fibers. Secondary injury, on the other hand, occurs as a result of a series of cascading reactions, such as oxidative stress, inflammatory response, neurotoxicity caused by Ca^2+^ and glutamate, which further deepen the extent of damage and expand the affected area (1). Among these reactions, oxidative stress is one of the important factors in the pathogenesis of secondary SCI (2). Currently, SCI is a challenging health problem worldwide. It has been reported that there are approximately 17,500 new cases of SCI in the United States annually, with the majority of patients experiencing severe clinical symptoms and complications, imposing significant physical, mental, and economic burdens on both the patients and their families (3). Due to the incomplete understanding of its pathogenesis, the treatment options for SCI are limited. Currently, early methylprednisolone combined with surgical intervention is the main approach (4). As the primary injury caused by direct trauma is unavoidable, there is an urgent clinical need for an effective treatment specifically targeting secondary injury.

**Figure 1:**
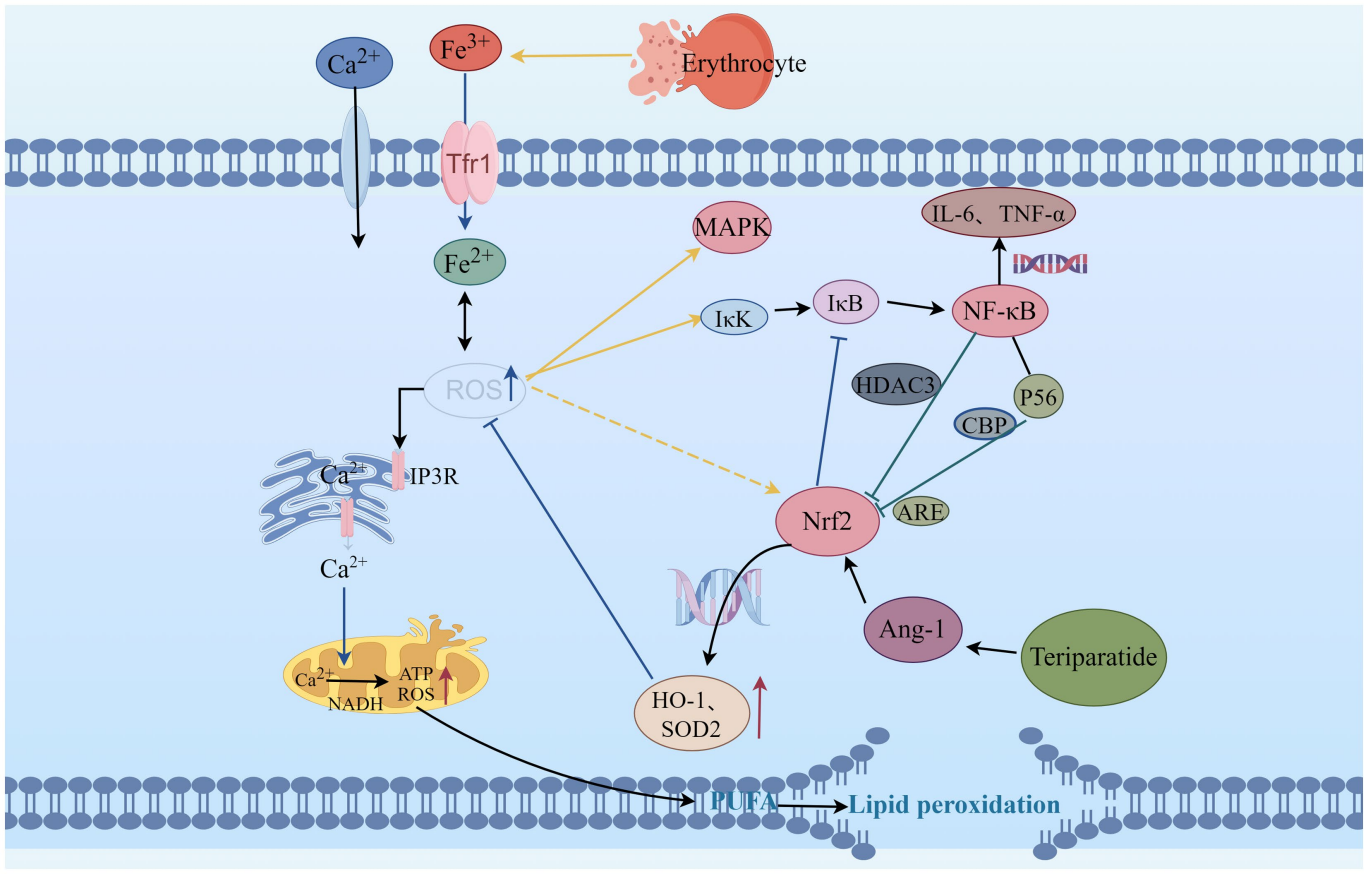
The relationship between Teriparatide and ROS. ROS, Reactive oxygen species; IP3R, inositol 1,4,5-trisphosphate receptors; MAPK, mitogen-activated protein kinase; Nrf2, Nuclear factor erythroid 2-related factor 2; HO-1, Heme Oxygenase-1; SOD2, Superoxide Dismutase 2; Trf1, Transferrin 1; NADH, Nicotinamide adenine dinucleotide; ATP, Adenosine Triphosphate; ARE, Antioxidant response elements; NF-κB, Nuclear factor kappa-B; IκB, inhibitor of NF-κB; IκK, activate IκB kinase; HDAC3, histone deacetylase 3; CBP, Cyclic-AMP response element-binding protein; P56, subunit of NF-κB; IL-6, Interleukin-6; TNF-α, Tumor necrosis factor-α; Ang-1, Angiopoietin-1; PUFA, Polyunsaturated fatty acids.

Teriparatide is a synthetically produced analog of the parathyroid hormone (PTH), also known as recombinant human PTH 1-34. It consists of the first 34 amino acid residues of the PTH molecule. Teriparatide is the first bone metabolism drug authorized by the U.S. Food and Drug Administration for the treatment of osteoporosis (5, 6). It not only has the function of regulating bone metabolism, but also can play a role in regulating oxidative stress (7). Study have shown that therapeutic doses of teriparatide can significantly reduce the production of reactive oxygen species (ROS) within the osteocytes of patients with osteoporosis, effectively inhibiting oxidative stress responses (7). Currently, this effect is also being investigated in the field of neuroscience (8), and it has promising experimental results. However, the evidence linking PTH and SCI mainly comes from scattered case reports (9–11), and the exact role of PTH in the SCI process remains unclear. This article aims to review the oxidative stress-related mechanisms following SCI and the connection with PTH, as well as to explore the potential therapeutic role of teriparatide in SCI (Figure 1).

## The relationship between SCI with oxidative stress

Oxidative stress refers to the imbalance between oxidation and antioxidant systems in the body, leading to the overproduction of ROS, reactive nitrogen species, and other excessive free radicals that exert toxic effects (12). Free radicals derived from O_2_ include O^2−^, OH^−^, and others, collectively referred to as ROS (13). Free radicals are normal byproducts of mitochondrial oxidative metabolism in the human body. During mitochondrial oxidative respiration, molecules with unpaired electrons, namely free radicals, are formed. The unpaired electrons make free radicals unstable and prone to react with other molecules, such as proteins, lipids, and DNA (14, 15).

Oxidative stress is one of the important aspects of the secondary pathological process of SCI (16). Excessive free radicals are produced due to ischemia and hypoxia during SCI, and the body’s own antioxidants are depleted within days or even hours after SCI (17). The generated antioxidants are insufficient to counteract the oxidative stress caused by SCI, and the excess oxidants cause irreversible damage to nerve cells (12, 15). This leads to nerve cell death and tissue damage (18). Increasing evidence suggests that reducing the production of ROS after SCI can effectively protect nerve cells from the effects of oxidative stress, mitigate the pathological process of spinal cord damage, promote neuronal repair and axonal regeneration (19, 20).

The intracellular homeostasis is disrupted after SCI, characterized by increased excitotoxicity of glutamate, elevated levels of free iron, and increased membrane permeability of Ca^2+^ (21). The elevated intracellular Ca^2+^ levels activate the nicotinamide adenine dinucleotide, which promotes ATP production while also increasing the production of ROS (22, 23). Excessive ROS can increase the permeability of Ca^2+^ membranes, leading to an increased influx of Ca^2+^, which causes a decrease in membrane potential. This activates the mitochondrial autophagy signaling pathway, inducing mitochondrial autophagy and further releasing excessive ROS, forming a vicious cycle (24). Recent study by Han et al. (25) have demonstrated that enhancing mitochondrial transport after SCI helps remove damaged mitochondria and replenish injured axons with normal mitochondria, thereby restoring local mitochondrial integrity and enhancing local ATP supply to meet the energy metabolic demands of axonal regeneration. This activates the intrinsic “growth program” in the central nervous system, promoting axonal regeneration and functional recovery after injury. Additionally, Ca^2+^ overload can enhance the activity of protein kinases and phospholipases, inducing protein degradation and lipid oxidation damage (26).

Due to the high content of polyunsaturated fatty acids in the spinal cord, oxygen free radicals can promote the generation of peroxidation intermediates such as 4-hydroxynonenal and malondialdehyde during SCI, leading to lipid oxidation degradation and disruption of the lipid bilayer structure of cell membranes (27, 28). The oxidative phospholipid markers in the core region of spinal cord lesions are significantly increased, it exacerbates the pathological process of SCI (29). The ROS scavengers can reduce lipid peroxidation induced by SCI, mitigate tissue damage, and improve neurological function (30). Moreover, the internal environment after SCI is in an acidic state, mechanical forces cause blood vessel rupture and bleeding, erythrocyte rupture, consequently free iron is increased (31). Neurons take up iron through transferrin and transferrin receptor-mediated iron uptake, leading to intracellular iron overload (32). Ferrous ions can participate in the aforementioned lipid peroxidation through Fenton reaction, generating a large amount of ROS, thereby causing secondary lipid peroxidation and further exacerbating oxidative stress (33). ROS can also react with C-H, S-H, N-H, or O-H in proteins, mediating the cleavage of peptide chains and modification of amino acid side chains, leading to protein misfolding, altered protein function, and increased susceptibility to hydrolysis and degradation (34). Experimental study (35) have shown that after SCI, proteins in the spinal cord are easily oxidized by oxidants to form advanced oxidation protein products. It can induce the expression of ROS through nicotinamide adenine dinucleotide phosphate oxidase, and excessive ROS can activate mitogen-activated protein kinase (MAPK) and nuclear factor kappa-B (NF-κB), inducing neuronal apoptosis.

Nuclear factor E2-related factor 2 (Nrf2)/Antioxidant Response Element (ARE) is an endogenous antioxidant defense mechanism of the body. It not only regulates oxidative stress but also participates in the regulation of inflammatory signaling pathways such as NF-κB and MAPK (36). Under normal physiological conditions, Nrf2 can form a dimer with Kelch-like ECH-associated protein 1 (Keap1) in the cytoplasm, resulting in the inhibition of Nrf2 activity (37). The Nrf2/ARE signaling pathway plays an important neuroprotective role in the early stage of SCI (38). After being activated by ROS, the Nrf2-Keap1 dimer dissociates, leading to Nrf2 phosphorylation and translocation into the cell nucleus, inducing the expression of a series of antioxidant enzyme genes such as heme oxygenase-1 (HO-1), NAD(P) H quinone oxidoreductase 1 (NQO1), and superoxide dismutase (SOD), directly or indirectly clearing free radicals, thus reducing or eliminating ROS, reducing cell oxidative stress (39). When the Keap1 gene is knocked out (40), Nrf2 is persistently activated in SCI astrocytes, which can increase the expression of NQO1, inhibit oxidative stress responses, reduce the damage to myelin and myelin-associated proteins, effectively protect neurons, and improve neurological functions. Study have shown (41) that upregulating Nrf2 expression and promoting HO-1 generation with polysaccharides effectively inhibits ROS production, suppresses oxidative stress response after SCI, and reduces neuronal apoptosis. This phenomenon can be reversed when siRNA silences Nrf2 expression.

Under physiological conditions, Nrf2 and nuclear factor kappa-B (NF-κB) signaling pathways are mutually coordinated to maintain cellular homeostasis (42). Under pathological conditions, oxidative stress can mediate the occurrence of inflammatory reactions, and excessive ROS can activate IκB kinase (IκK), inducing phosphorylation of its inhibitor IκB and leading to proteasomal degradation of IκB protein. This results in nuclear translocation of NF-κB and activation of downstream genes such as IL-6 and TNF-α (43). Nrf2 can inhibit the degradation of IκB-α, thereby blocking NF-κB nuclear translocation and the transcription of pro-inflammatory genes (42). Conversely, NF-κB can enhance the recruitment of histone deacetylase 3 to the antioxidant response element region, leading to inhibition of Nrf2 activity and hindrance of ARE gene transcription, thereby suppressing antioxidant responses (44). P65 is a subunit of NF-κB, and ARE is the gene binding site of Nrf2. p65 has the ability to inhibit the expression of ARE genes (45), and on the other hand, the p65 subunit can also bind with the CREB binding protein (CBP), a transcriptional co-activator of Nrf2, to jointly compete for the CH1-KIX domain of CBP, thereby inhibiting the Nrf2 signaling pathway (46). Research has found (47) that during SCI, experimental drug intervention can upregulate the expression of Nrf2 and inhibit the release of NF-κB-related mediators. When Nrf2 is suppressed, the expression of NF-κB-related factors increases, revealing that Nrf2 can be involved in regulating the NF-κB signaling pathway after SCI, thereby reducing neuronal death. Li et al. (48) found that inhibiting the expression of HO-1 can increase in NF-κB-related factors. Based on these findings, it is possible that Nrf2 and NF-κB may interact and influence each other in the oxidative stress response following SCI.

## The correlation between PTH and SCI

Vaziri et al. (49) first reported the association between PTH and SCI. By examining various laboratory parameters in the serum of 40 SCI patients, they found a decrease in PTH levels after SCI, and significantly lower PTH levels in the SCI group compared to the control group, suggesting a potential correlation between PTH and SCI. Subsequently, Mechanick et al. (9) divided SCI patients into complete paralysis and incomplete paralysis groups according to the ASIA classification. They found a more pronounced decrease in PTH levels in the complete paralysis group, leading the authors to speculate that the severity of neural damage is related to the reduction of PTH. In recent years, Ouyang et al. (50) conducted further clinical research on the relationship between PTH and SCI, revealing a decrease in peripheral blood PTH levels in SCI patients and a significant correlation with the severity of SCI.

Similar phenomena have also been observed in animal models. Rouleau et al. (10) found that the levels of PTH in the serum of mice with SCI remained low throughout the entire animal experiment, with the most significant decrease occurring within 1 week after injury. This suggests that PTH synthesis and metabolism are related to the immune system after SCI. Del et al. (11) also found that PTH gradually decreased over time in mice with SCI, further demonstrating that PTH is one of the pathological and physiological factors affecting SCI. Subsequent studies (51, 52) have focused more on studying the effects of PTH on bone structure and bone density after SCI, as well as research related to osteoporosis and fracture healing involving PTH replacement therapies such as teriparatide (Table 1). However, they have not further explored whether it can promote the repair of neurological function after SCI. Recent study (8) have found that teriparatide can activate the Nrf2 pathway by inducing the production of angiogenin 1, which in turn increases the expression of its downstream antioxidant proteins HO-1 and SOD2, inhibit oxidative stress and improve nerve function. However, the exact mechanism of action has not been further studied by the authors. Therefore, in the early stages of SCI, teriparatide may exert its pharmacological effects by targeting neuronal cells.

**Table 1:** Correlation between PTH and spinal cord injury.

Study	Time	Research object	Results
Vaziri et al. (49)	1994	Human	SCI: decrease in PTH
Mechanick et al. (9)	1997	Human	PTH related to ASIA
Ouyang et al. (50)	2021	Human	PTH related to the degree of SCI
Rouleau et al. (10)	2007	Mouse	SCI: PTH decreased significantly within 1 week, and related to the immune system
Del et al. (11)	2016	Mouse	SCI: decrease in PTH
Sahbani et al. and Le et al. (51, 52)	2019 and 2021	Human	Decrease in PTH, teriparatide treat osteoporosis after SCI.

SCI, Spinal cord injury; PTH, Parathyroid hormone; ASIA, American Spinal Injury Association Impairment Scale, International Standards for Neurological Classification of Spinal Cord Injury.

## The role of teriparatide in oxidative stress

**Figure 2:**
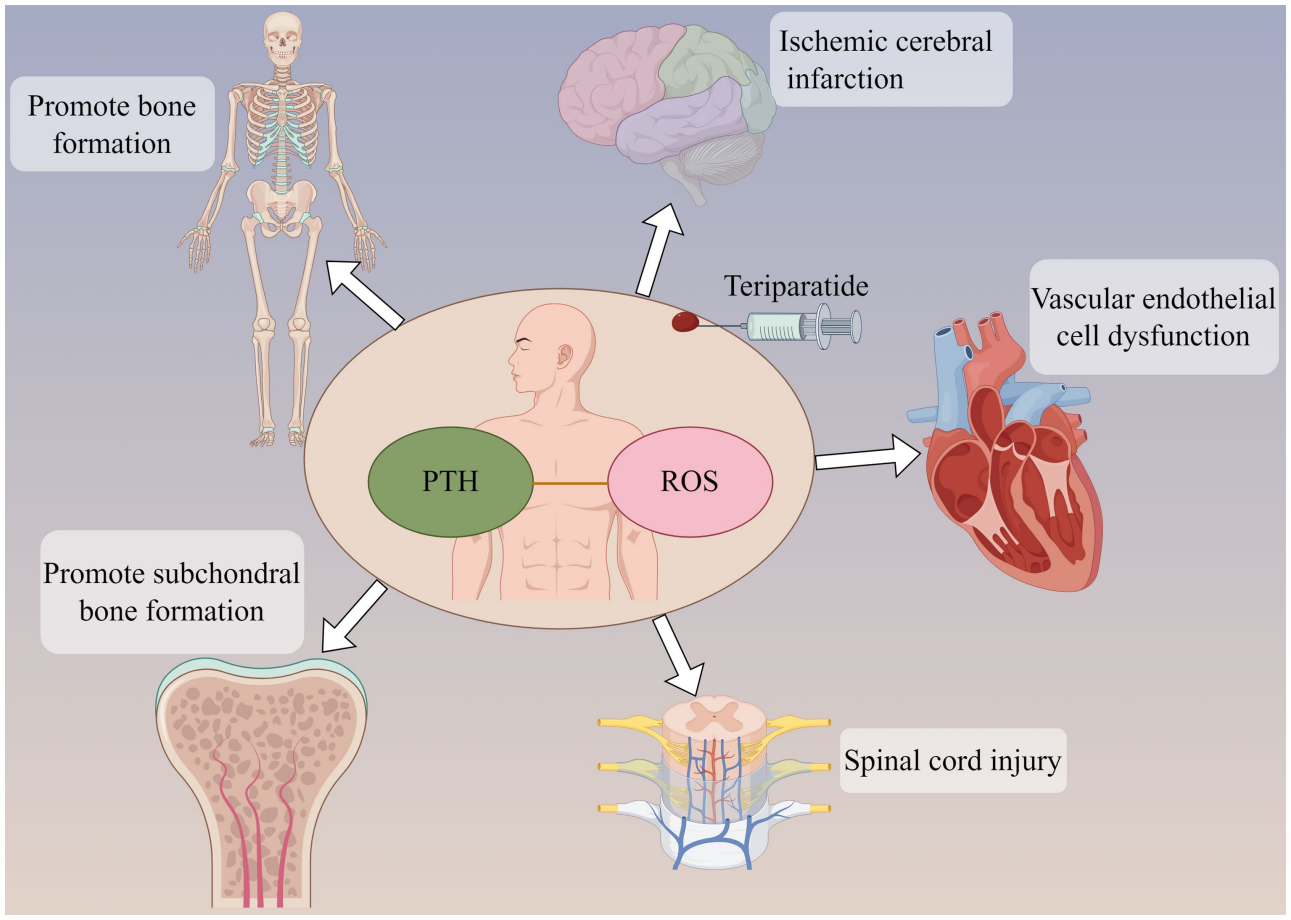
Parathyroid hormone (PTH) can not only promote bone formation and subchondral bone formation, but also act on ischemic cerebral infarction and reduce brain tissue injury. PTH may be involved in the pathological development of cardiovascular disease and spinal cord injury. The participation of PTH may be related to oxidative stress response in the above effects.

The effects of PTH vary in different tissues. In endothelial cells, PTH acts on surface receptors to generate IP3, leading to an increase in intracellular Ca^2+^ concentration. Excessive calcium uptake by mitochondria subsequently increases the production of ROS, thereby inducing oxidative stress responses, which result in endothelial cell dysfunction (53). Research has discovered that PTH can activate DNA repair proteins (nuclear antigens) and FOXO transcription factor 3a, downregulate DNA damage protein 153, thereby enabling cells to avoid DNA damage caused by oxidative stress (54). Additionally, in osteoporosis induced by dexamethasone, dexamethasone can lead to an increase in ROS within bone cells. Concurrent use of teriparatide intervention while stimulating bone cells with dexamethasone can reduce the production of intracellular ROS, mitigate the harmful effects of dexamethasone on bone cells, promote the proliferation of bone cells, and prevent the occurrence of osteoporosis (7). Ardura (55) found that PTH analogs can increase the phosphorylation of Extracellular regulated protein kinases and Protein kinase B, regulate MAPK phosphatase-1 to activate MAPK dephosphorylation, enhance expression of peroxidase genes, reduce ROS production, suppress oxidative stress response, and decrease cell apoptosis (Figure 2).

Parathyroid hormone-related peptide (PTHrP) is a highly bioactive small molecule peptide that, in some respects, possesses superior functions to PTH (1–34). Its structure and mechanism of action are not entirely identical to those of PTH (1–34) (56). Study (57) have shown that PTHrp can reduce ROS production in mesenchymal stem cells and stimulate cartilage formation. Wang et al. (58) discovered that PTHrP can attenuate the differentiation of osteoclast-like cells. Even under an oxidative stress environment, PTHrP is capable of protecting mesenchymal stem cells and human umbilical vein endothelial cells by reducing the production of ROS and mitochondrial damage, thereby promoting proliferation, migration, and angiogenesis. Therefore, PTH and its analogs play a role in regulating oxidative stress (Table 2).

**Table 2:** Relationship between PTH and oxidative stress in disease.

Disease	Effect	Main result	References
Cardiovascular disease	Negative	PTH: increases in ROS	(53)
Osteoporosis disease	Positive	Teriparatide: decrease in ROS	(7, 54)
	Positive	PTHrP: decrease in ROS	(55, 58)
Osteoarthritis	Positive	PTHrp: decrease in ROS	(57)
Ischemic cerebral infarction	Positive	Teriparatide: decrease in ROS	(8)

PTH, parathyroid hormone; ROS, Reactive oxygen species; PTHrP, PTH derivative; Teriparatide, new recombinant human parathyroid hormone.

## Summary and perspective

In summary, SCI is associated with oxidative stress mediated by the Nrf2 and NF-κB cascades. As an Nrf2 activator, teriparatide may be used to treat SCI by targeting this pathway. However, current research on teriparatide in SCI mainly focuses on bone metabolism, and there are no reports on its effects on neurological function. Therefore, further validation is needed to determine whether teriparatide affects the pathological mechanisms of SCI through the Nrf2 signaling pathway.

## Author contributions

GA: Conceptualization, Data curation, Formal analysis, Investigation, Methodology, Software, Writing – original draft. MX: Conceptualization, Methodology, Software, Supervision, Writing – review & editing. LD: Methodology, Software, Supervision, Validation, Writing – review & editing. JZ: Investigation, Resources, Supervision, Visualization, Writing – review & editing. QX: Conceptualization, Project administration, Resources, Validation, Writing – review & editing.

## References

[1] McDonald JW Sadowsky C. Spinal-cord injury. Lancet. (2002) 359:417–25. doi: 10.1016/S0140-6736(02)07603-1 11844532

[2] Li Z Zhao T Ding J Gu H Wang Q Wang Y. A reactive oxygen species-responsive hydrogel encapsulated with bone marrow derived stem cells promotes repair and regeneration of spinal cord injury. Bioact Mater. (2023) 19:550–68. doi: 10.1016/j.bioactmat.2022.04.029, PMID: 35600969 PMC9108756

[3] Schwab JM Maas A Hsieh J Curt A. Raising awareness for spinal cord injury research. Lancet Neurol. (2018) 17:581–2. doi: 10.1016/S1474-4422(18)30206-0 29914704

[4] Lambrechts MJ Issa TZ Hilibrand AS. Updates in the early Management of Acute Spinal Cord Injury. J Am Acad Orthop Surg. (2023) 31:e619–32. doi: 10.5435/JAAOS-D-23-00281, PMID: 37432977

[5] Leder BZ Tsai JN Uihlein AV Wallace PM Lee H Neer RM. Denosumab and teriparatide transitions in postmenopausal osteoporosis (the DATA-switch study): extension of a randomised controlled trial. Lancet. (2015) 386:1147–55. doi: 10.1016/S0140-6736(15)61120-5, PMID: 26144908 PMC4620731

[6] Bashutski JD Eber RM Kinney JS Benavides E Maitra S Braun TM. Teriparatide and osseous regeneration in the oral cavity. N Engl J Med. (2010) 363:2396–405. doi: 10.1056/NEJMoa1005361, PMID: 20950166 PMC5695223

[7] Wang T Han C Tian P Li PF Ma XL. Role of Teriparatide in glucocorticoid-induced osteoporosis through regulating cellular reactive oxygen species. Orthop Surg. (2018) 10:152–9. doi: 10.1111/os.12369, PMID: 29745033 PMC6594530

[8] Xiong M Feng Y Huang S Lv S Deng Y Li M. Teriparatide induces angiogenesis in ischemic cerebral infarction zones of rats through AC/PKA signaling and reduces ischemia-reperfusion injury. Biomed Pharmacother. (2022) 148:112728. doi: 10.1016/j.biopha.2022.112728, PMID: 35220030

[9] Mechanick JI Pomerantz F Flanagan S Stein A Gordon WA Ragnarsson KT. Parathyroid hormone suppression in spinal cord injury patients is associated with the degree of neurologic impairment and not the level of injury. Arch Phys Med Rehabil. (1997) 78:692–6. doi: 10.1016/S0003-9993(97)90075-7, PMID: 9228870

[10] Rouleau P Ung RV Lapointe NP Guertin PA. Hormonal and immunological changes in mice after spinal cord injury. J Neurotrauma. (2007) 24:367–78. doi: 10.1089/neu.2006.0117, PMID: 17376000

[11] Del RT Bethea JR. The effects of spinal cord injury on bone loss and dysregulation of the calcium/parathyroid hormone loop in mice. Osteoporos Sarcopenia. (2016) 2:164–9. doi: 10.1016/j.afos.2016.06.003 30775482 PMC6372742

[12] Forman HJ Zhang H. Targeting oxidative stress in disease: promise and limitations of antioxidant therapy. Nat Rev Drug Discov. (2021) 20:689–709. doi: 10.1038/s41573-021-00233-1, PMID: 34194012 PMC8243062

[13] Sies H. Oxidative eustress: on constant alert for redox homeostasis. Redox Biol. (2021) 41:101867. doi: 10.1016/j.redox.2021.101867, PMID: 33657525 PMC7930632

[14] Foo J Bellot G Pervaiz S Alonso S. Mitochondria-mediated oxidative stress during viral infection. Trends Microbiol. (2022) 30:679–92. doi: 10.1016/j.tim.2021.12.011, PMID: 35063304

[15] Nolfi-Donegan D Braganza A Shiva S. Mitochondrial electron transport chain: oxidative phosphorylation, oxidant production, and methods of measurement. Redox Biol. (2020) 37:101674. doi: 10.1016/j.redox.2020.101674, PMID: 32811789 PMC7767752

[16] Visavadiya NP Patel SP VanRooyen JL Sullivan PG Rabchevsky AG. Cellular and subcellular oxidative stress parameters following severe spinal cord injury. Redox Biol. (2016) 8:59–67. doi: 10.1016/j.redox.2015.12.011, PMID: 26760911 PMC4712315

[17] Zhou K Zheng Z Li Y Han W Zhang J Mao Y. TFE3, a potential therapeutic target for spinal cord injury via augmenting autophagy flux and alleviating ER stress. Theranostics. (2020) 10:9280–302. doi: 10.7150/thno.46566, PMID: 32802192 PMC7415792

[18] Nukolova NV Aleksashkin AD Morozova AY Gubskiy IL Kirzhanova ЕА Abakumov MA. Multilayer polyion complex nanoformulations of superoxide dismutase 1 for acute spinal cord injury. J Control Release. (2018) 270:226–36. doi: 10.1016/j.jconrel.2017.11.044, PMID: 29196042

[19] Ji ZS Gao GB Ma YM Luo JX Zhang GW Yang H. Highly bioactive iridium metal-complex alleviates spinal cord injury via ROS scavenging and inflammation reduction. Biomaterials. (2022) 284:121481. doi: 10.1016/j.biomaterials.2022.121481, PMID: 35405576

[20] Andrabi SS Yang J Gao Y Kuang Y Labhasetwar V. Nanoparticles with antioxidant enzymes protect injured spinal cord from neuronal cell apoptosis by attenuating mitochondrial dysfunction. J Control Release. (2020) 317:300–11. doi: 10.1016/j.jconrel.2019.12.001, PMID: 31805339 PMC7007870

[21] Liu X Jiang X Yu Q Shen W Tian H Mei X. Sodium alginate and naloxone loaded macrophage-derived nanovesicles for the treatment of spinal cord injury. Asian J Pharm Sci. (2022) 17:87–101. doi: 10.1016/j.ajps.2021.11.001, PMID: 35261646 PMC8888181

[22] Huang Y Wang J Yue C Wang R Guo Q Wang T. An in situ assembled trapping gel repairs spinal cord injury by capturing glutamate and free calcium ions. Small. (2023) 19:e2206229. doi: 10.1002/smll.202206229, PMID: 36683214

[23] Neves D Salazar IL Almeida RD Silva RM. Molecular mechanisms of ischemia and glutamate excitotoxicity. Life Sci. (2023) 328:121814. doi: 10.1016/j.lfs.2023.121814 37236602

[24] Slater PG Dominguez-Romero ME Villarreal M Eisner V Larrain J. Mitochondrial function in spinal cord injury and regeneration. Cell Mol Life Sci. (2022) 79:239. doi: 10.1007/s00018-022-04261-x 35416520 PMC11072423

[25] Han Q Xie Y Ordaz JD Huh AJ Huang N Wu W. Restoring cellular energetics promotes axonal regeneration and functional recovery after spinal cord injury. Cell Metab. (2020) 31:623–641.e8. doi: 10.1016/j.cmet.2020.02.002, PMID: 32130884 PMC7188478

[26] Xu X Wang X Yang Y Ares I Martinez M Lopez-Torres B. Neonicotinoids: mechanisms of systemic toxicity based on oxidative stress-mitochondrial damage. Arch Toxicol. (2022) 96:1493–520. doi: 10.1007/s00204-022-03267-5, PMID: 35344072

[27] Dong Y Yong VW. Oxidized phospholipids as novel mediators of neurodegeneration. Trends Neurosci. (2022) 45:419–29. doi: 10.1016/j.tins.2022.03.002, PMID: 35393134

[28] Hill RL Singh IN Wang JA Kulbe JR Hall ED. Protective effects of phenelzine administration on synaptic and non-synaptic cortical mitochondrial function and lipid peroxidation-mediated oxidative damage following TBI in young adult male rats. Exp Neurol. (2020) 330:113322. doi: 10.1016/j.expneurol.2020.113322, PMID: 32325157 PMC7418938

[29] Zrzavy T Schwaiger C Wimmer I Berger T Bauer J Butovsky O. Acute and non-resolving inflammation associate with oxidative injury after human spinal cord injury. Brain. (2021) 144:144–61. doi: 10.1093/brain/awaa360, PMID: 33578421 PMC7880675

[30] Gao L Zhang Z Xu W Li T Ying G Qin B. Natrium benzoate alleviates neuronal apoptosis via the DJ-1-related anti-oxidative stress pathway involving Akt phosphorylation in a rat model of traumatic spinal cord injury. Front Mol Neurosci. (2019) 12:42. doi: 10.3389/fnmol.2019.00042 30853891 PMC6395451

[31] Chio J Xu KJ Popovich P David S Fehlings MG. Neuroimmunological therapies for treating spinal cord injury: evidence and future perspectives. Exp Neurol. (2021) 341:113704. doi: 10.1016/j.expneurol.2021.113704, PMID: 33745920

[32] Hu X Xu Y Xu H Jin C Zhang H Su H. Progress in understanding Ferroptosis and its targeting for therapeutic benefits in traumatic brain and spinal cord injuries. Front Cell Dev Biol. (2021) 9:705786. doi: 10.3389/fcell.2021.705786, PMID: 34422826 PMC8371332

[33] Anjum A Yazid MD Fauzi DM Idris J Ng A Selvi NA. Spinal cord injury: pathophysiology, multimolecular interactions, and underlying recovery mechanisms. Int J Mol Sci. (2020) 21:533. doi: 10.3390/ijms21207533, PMID: 33066029 PMC7589539

[34] Yu M Wang Z Wang D Aierxi M Ma Z Wang Y. Oxidative stress following spinal cord injury: from molecular mechanisms to therapeutic targets. J Neurosci Res. (2023) 101:1538–54. doi: 10.1002/jnr.25221, PMID: 37272728

[35] Liu Z Yao X Jiang W Li W Zhu S Liao C. Advanced oxidation protein products induce microglia-mediated neuroinflammation via MAPKs-NF-kappaB signaling pathway and pyroptosis after secondary spinal cord injury. J Neuroinflammation. (2020) 17:90. doi: 10.1186/s12974-020-01751-2, PMID: 32192500 PMC7082940

[36] Guo X Kang J Wang Z Wang Y Liu M Zhu D. Nrf2 signaling in the oxidative stress response after spinal cord injury. Neuroscience. (2022) 498:311–24. doi: 10.1016/j.neuroscience.2022.06.007, PMID: 35710066

[37] Cano M Datta S Wang L Liu T Flores-Bellver M Sachdeva M. Nrf2 deficiency decreases NADPH from impaired IDH shuttle and pentose phosphate pathway in retinal pigmented epithelial cells to magnify oxidative stress-induced mitochondrial dysfunction. Aging Cell. (2021) 20:e13444. doi: 10.1111/acel.13444, PMID: 34313391 PMC8373343

[38] Ebrahimy N Gasterich N Behrens V Amini J Fragoulis A Beyer C. Neuroprotective effect of the Nrf2/ARE/miRNA145-5p signaling pathway in the early phase of spinal cord injury. Life Sci. (2022) 304:120726. doi: 10.1016/j.lfs.2022.120726, PMID: 35750202

[39] Jiang T He Y. Recent advances in the role of nuclear factor Erythroid-2-related factor 2 in spinal cord injury: regulatory mechanisms and therapeutic options. Front Aging Neurosci. (2022) 14:851257. doi: 10.3389/fnagi.2022.851257, PMID: 35754957 PMC9226435

[40] Zhao W Gasterich N Clarner T Voelz C Behrens V Beyer C. Astrocytic Nrf2 expression protects spinal cord from oxidative stress following spinal cord injury in a male mouse model. J Neuroinflammation. (2022) 19:134. doi: 10.1186/s12974-022-02491-1, PMID: 35668451 PMC9169394

[41] Lv R Du L Zhang L Zhang Z. Polydatin attenuates spinal cord injury in rats by inhibiting oxidative stress and microglia apoptosis via Nrf2/HO-1 pathway. Life Sci. (2019) 217:119–27. doi: 10.1016/j.lfs.2018.11.053, PMID: 30481506

[42] Casper E. The crosstalk between Nrf2 and NF-kappaB pathways in coronary artery disease: can it be regulated by SIRT6? Life Sci. (2023) 330:122007. doi: 10.1016/j.lfs.2023.122007, PMID: 37544377

[43] Sivandzade F Prasad S Bhalerao A Cucullo L. NRF2 and NF-κB interplay in cerebrovascular and neurodegenerative disorders: molecular mechanisms and possible therapeutic approaches. Redox Biol. (2019) 21:101059. doi: 10.1016/j.redox.2018.11.017, PMID: 30576920 PMC6302038

[44] Hayes JD Dinkova-Kostova AT Tew KD. Oxidative stress in Cancer. Cancer Cell. (2020) 38:167–97. doi: 10.1016/j.ccell.2020.06.001, PMID: 32649885 PMC7439808

[45] Atalay ES Gegotek A Skrzydlewska E. The molecular activity of cannabidiol in the regulation of Nrf2 system interacting with NF-kappaB pathway under oxidative stress. Redox Biol. (2022) 57:102489. doi: 10.1016/j.redox.2022.102489, PMID: 36198205 PMC9535304

[46] Gao W Guo L Yang Y Wang Y Xia S Gong H. Dissecting the crosstalk between Nrf2 and NF-kappaB response pathways in drug-induced toxicity. Front Cell Dev Biol. (2021) 9:809952. doi: 10.3389/fcell.2021.809952 35186957 PMC8847224

[47] Xia M Zhang Y Wu H Zhang Q Liu Q Li G. Forsythoside B attenuates neuro-inflammation and neuronal apoptosis by inhibition of NF-kappaB and p38-MAPK signaling pathways through activating Nrf2 post spinal cord injury. Int Immunopharmacol. (2022) 111:109120. doi: 10.1016/j.intimp.2022.109120, PMID: 35944463

[48] Li Z Wu F Xu D Zhi Z Xu G. Inhibition of TREM1 reduces inflammation and oxidative stress after spinal cord injury (SCI) associated with HO-1 expressions. Biomed Pharmacother. (2019) 109:2014–21. doi: 10.1016/j.biopha.2018.08.159, PMID: 30551457

[49] Vaziri ND Pandian MR Segal JL Winer RL Eltorai I Brunnemann S. Vitamin D, parathormone, and calcitonin profiles in persons with long-standing spinal cord injury. Arch Phys Med Rehabil. (1994) 75:766–9. doi: 10.1016/0003-9993(94)90133-3, PMID: 8024422

[50] Ouyang YF Su J Guo X Wang LR He MR Yuan CY. Correlation analysis of pNF-H, S100B, bone gamma-carboxyglutamic-acid-containing proteins, parathyroid hormone and bone-specific alkaline phosphatase expression in peripheral blood of patients with spinal fracture and spinal cord injury and their degree of disease. Chinese J Exp Surg. (2021) 38:2044. doi: 10.3760/cma.j.cn421213-20210618-00475

[51] Sahbani K Cardozo CP Bauman WA Tawfeek HA. Abaloparatide exhibits greater osteoanabolic response and higher cAMP stimulation and beta-arrestin recruitment than teriparatide. Physiol Rep. (2019) 7:e14225. doi: 10.14814/phy2.14225, PMID: 31565870 PMC6766518

[52] Le B Ray C Gonzalez B Miskevics S Weaver FM Priebe M. Reasons for initiation and discontinuation of pharmacological therapies for osteoporosis in veterans with spinal cord injury and disorders. J Clin Densitom. (2021) 24:67–77. doi: 10.1016/j.jocd.2019.06.003, PMID: 31262561

[53] Gambardella J De Rosa M Sorriento D Prevete N Fiordelisi A Ciccarelli M. Parathyroid hormone causes endothelial dysfunction by inducing mitochondrial ROS and Specific oxidative signal transduction modifications. Oxidative Med Cell Longev. (2018) 2018:9582319. doi: 10.1155/2018/9582319 PMC6313989 30662585

[54] Schnoke M Midura SB Midura RJ. Parathyroid hormone suppresses osteoblast apoptosis by augmenting DNA repair. Bone. (2009) 45:590–602. doi: 10.1016/j.bone.2009.05.006, PMID: 19450716 PMC2752836

[55] Ardura JA Portal-Nunez S Castelbon-Calvo I Martinez DTI De la Fuente M Esbrit P. Parathyroid hormone-related protein protects osteoblastic cells from oxidative stress by activation of MKP1 phosphatase. J Cell Physiol. (2017) 232:785–96. doi: 10.1002/jcp.25473, PMID: 27357344

[56] Martin TJ Sims NA Seeman E. Physiological and pharmacological roles of PTH and PTHrP in bone using their shared receptor, PTH1R. Endocr Rev. (2021) 42:383–406. doi: 10.1210/endrev/bnab005, PMID: 33564837

[57] Yang Y Lei H Wang B. Effect of the PTHrP(1-34) analog abaloparatide on inducing chondrogenesis involves inhibition of intracellular reactive oxygen species production. Biochem Biophys Res Commun. (2019) 509:960–5. doi: 10.1016/j.bbrc.2019.01.049, PMID: 30654932 PMC7768806

[58] Wang J Chen R Ren B Feng Q Li B Hao Z. A novel PTH‐related peptide combined with 3D printed macroporous titanium alloy scaffold enhances osteoporotic Osseointegration. Adv Healthc Mater. (2023) 12:e2301604. doi: 10.1002/adhm.202301604 37584445

